# Augmentative and alternative communication in autism spectrum disorder: transitioning from letter board to iPad – a case study

**DOI:** 10.3389/fpsyt.2024.1345447

**Published:** 2024-05-21

**Authors:** Dionisia Mavritsakis

**Affiliations:** Royal College of Surgeons in Ireland, School of Medicine, Dublin, Ireland

**Keywords:** autism spectrum disorder (ASD), communication and language, augmentative and alternative communication (AAC), cognitive abilities, apraxia

## Abstract

This case study examines the effective use of Augmentative and Alternative Communication (AAC) tools in enhancing communication skills in a 15-year-old male with Autism Spectrum Disorder (ASD). Initially exhibiting non-verbal tendencies, the subject experienced significant improvements in communication and social interaction following the introduction of a letter board and subsequent transition to an iPad. These AAC tools facilitated a marked development in his ability to articulate thoughts, engage in academic activities, and express complex ideas, particularly in science. The study highlights the role of AAC in expanding the communicative capabilities of individuals with limited or no verbal language, demonstrating notable advancements in both verbal expression and cognitive engagement. The findings underscore the transformative impact of personalized AAC interventions and suggest the potential for broader application in ASD support strategies. This case highlights the need for further research, including randomized controlled trials, to explore the efficacy of AAC tools in diverse ASD contexts.

## Introduction

Autism Spectrum Disorder (ASD) is a neurodevelopmental disorder characterized by a spectrum of communication challenges, notably tendencies toward non-verbal communication (1). Timely intervention and efficacious communication methodologies are imperative for individuals diagnosed with ASD to realize their optimal developmental outcomes. Augmentative and Alternative Communication (AAC) devices, encompassing letter boards and digital platforms like iPads, have emerged as promising tools to bolster communication in individuals with limited or no verbal language. In this case study, we delineate the developmental trajectory of a child diagnosed with ASD, who grappled with pronounced communication impediments during his formative years. These years were marked by challenges in verbalizing thoughts and emotions. Notwithstanding these challenges, consistent and dedicated support from his familial environment, particularly from his father, an eminent orthopedic surgeon, proved pivotal in facilitating his developmental milestones. This supportive framework culminated in the introduction of a letter board as an AAC modality, heralding the onset of his transformative communicative journey. Subsequently, a transition to a more sophisticated AAC device, the iPad, was observed, which further amplified his communicative competencies.

## AAC’s role in combating stigma associated with non-verbal autism

Following the adoption of AAC tools in therapeutic practices, there is a need to address the stigma commonly associated with autism, particularly for individuals with limited or no verbal language. Misconceptions about these individuals frequently lead to underestimating their abilities and overlooking their potentials. This stigma not only affects the personal dignity of those individuals with ASD but also limits their access to tools that can help them communicate and interact with the world around them. Stigma stems from a lack of understanding and awareness about the capabilities of individuals with limited or no verbal language and manifests in educational, social, and clinical settings where these individuals are often prematurely judged to have lower intellectual capacities. This societal bias can hinder the provision and effectiveness of interventions like AAC tools, as caregivers, educators, and peers might not fully commit to the consistent use of these technologies. AAC devices challenge these stigmas by demonstrating the communicative competencies of individuals with limited or no verbal language. Through the case of this young male with ASD, we observe a significant transformation once AAC tools are integrated into his daily interactions. Initially perceived as having limited potential due to his challenges with traditional verbal communication, the introduction of a letter board and later an iPad revealed his remarkable ability to express complex thoughts and actively participate in his educational activities.

These tools not only facilitated his communication but also allowed his cognitive abilities and depth of understanding to become apparent, challenging previous misconceptions about his capabilities. These developments not only changed his communicative abilities but also altered the perceptions of those around him. By enabling expression of thoughts, AAC tools provide a platform for individuals with limited or no verbal language to participate more fully in social and academic environments, challenging preconceived notions about their limitations. The visibility of such interactions serves as a powerful testament to the potential residing within these individuals, advocating for a shift in societal attitudes and policies towards greater inclusivity and support. The successful integration of AAC tools into the life of the subject in this study highlights the need for systemic changes in how society views autistic people with limited or no verbal language. Educational systems, healthcare providers, and policy-makers are called upon to reconsider and reshape their approaches to autism, recognizing the crucial role of AAC tools in empowering these individuals. By integrating AAC devices from the onset of communication development, we improve the chances for these individuals to lead fulfilling and productive lives.

## Case description

In the early developmental stages, the subject exhibited restricted verbal communication because of severe dyspraxia both verbal and motor. However, a keen observation by the primary caregiver, his father, identified potential comprehension abilities, even when the subject’s expressions were non-traditional. This observation catalyzed the exploration of alternative communication modalities. Recognizing the subject’s intrinsic communication needs, an AAC device, specifically a letter board, was introduced, marking the commencement of his journey toward enhanced communication.

This simple yet effective tool employed was a cardboard plasticized letter board. This particular letter board was chosen for its straightforward design and ease of use, crucial for accommodating the subject’s motor skills and cognitive processing needs. The board featured a layout of large, clear letters, allowing the subject to point to each letter in sequence to spell out his thoughts and responses. The use of this letter board was a fundamental step in the subject’s communicative journey. It provided a tactile and visual means of expression, enabling him to construct sentences by pointing to letters in order. This method of spelling out speech was not only instrumental in facilitating basic communication but also played a significant role in enhancing his cognitive development. The repetitive action of pointing to letters and forming words helped in reinforcing his understanding of language and sentence structure. The simplicity of the letter board was key to its effectiveness as its basic design minimized distractions, allowing the subject to focus solely on the task of communication. This focus was essential in the early stages of his AAC journey, where the primary goal was to establish a reliable method of expressing his needs, thoughts, and emotions.

Initial engagements with the letter board were approached with optimism. Progressively, the subject achieved notable milestones in communication. Because of the severe dyspraxia, a lot of effort was required to help the subject develop the necessary coordination between eyes and his index finger in order to point to letters consistently. Anecdotal evidence underscores the depth of his ability to articulate and establish connections. The letter board functioned as a conduit to his cognitive processes. As the subject’s communication proficiency evolved, a transition to a more advanced AAC device, the iPad, was introduced. This transition expanded his communication accessibility and versatility. Notably, the subject exhibited a pronounced inclination toward science and advanced mathematics, utilizing the iPad as a primary tool using an external keypad rather than the glass keyboard. The device’s touchscreen interface facilitated an intuitive interaction. Its tactile feedback allowed for direct engagement with content, encompassing complex mathematical equations. The App Store offered a plethora of educational applications, augmenting his learning trajectory. The iPad’s integrated communication utilities, encompassing email and messaging applications, enabled consistent communication with the primary caregiver, educational facilitators, and peers. Significantly, the subject’s capability to address advanced mathematical problems underscores the profound efficacy of AAC devices. These findings not only demonstrate the transformative potential of AAC devices in enhancing communication for people with limited or no verbal language but also emphasize their capacity to empower subjects to excel in specialized domains.

## Familial and environmental support

A critical factor in the subject’s developmental journey was the support from his familial environment, particularly the role of his father, a surgeon, who was instrumental in identifying and pursuing the AAC approach. His father’s keen observation and medical background played a pivotal role in recognizing the potential of AAC tools and never giving up on the subject’s ability to communicate. This dedication was not just limited to selecting the appropriate communication tools but also extended to daily interactions and encouragement, fostering a supportive and nurturing environment for the subject’s growth. In addition to his father’s involvement, the subject benefited significantly from the presence of a shadow aide at school. This individual provided one-on-one assistance, ensuring that the subject could effectively use his AAC tools in the educational setting and facilitating his integration into mainstream education. The shadow aide acted as a bridge between the subject and his peers, as well as between the subject and his educators, playing a crucial role in his social and academic development. Furthermore, the subject’s much older sibling, with over a decade age gap, contributed significantly to his communicative journey. The sibling’s support complemented the father’s efforts, providing a different dimension of familial love and interaction. This sibling relationship was marked by a deep bond, with the older sibling consistently showing love and acceptance, further enriching the subject’s social and emotional experiences. The combined efforts of the father, shadow aide, and sibling created a comprehensive support system that was critical in the subject’s successful use of AAC tools and overall development. Their roles demonstrated the importance of a collaborative and holistic approach in supporting individuals with ASD, highlighting how family members and educational support can synergistically contribute to positive outcomes.

## Educational materials and reading approach

Contrary to creating customized educational materials, the father introduced his son to a range of advanced science and mathematics books, as well as fiction, catering to his interests and apparent photographic memory. The subject displayed a keen interest in adult-level educational books, particularly those about animals and other scientific topics, which were well above his age level. This approach was based on the observation that the child showed little interest in typical children’s books. The father’s method involved reading these advanced books together with his son and during these reading sessions, the father would ask questions about the content, to which the subject would respond using his letter board. This interactive reading approach not only catered to the subject’s interests but also significantly contributed to his cognitive development as it provided a platform for the subject to engage with complex material in a way that was both educational and stimulating. These reading sessions were more than just a learning exercise as they were a means of communication and connection between the father and son. The choice of material, far from being random, was carefully selected to align with the subject’s interests and abilities, thereby fostering an environment of learning that was both challenging and rewarding.

## Utilization of iPad applications for enhanced communication

In the realm of digital tools, the subject’s use of iPad applications was notably simplistic yet highly effective. The primary application used was a basic word prediction and completion tool, designed to expedite and streamline his written communication. This app functioned by suggesting words based on the initial letters input by the subject. For instance, upon typing “th,” the app would offer options like “the,” “them,” “there,” allowing the subject to quickly select the intended word. This straightforward application played a crucial role in enhancing the subject’s communication efficiency as it allowed him to express his thoughts more rapidly and with greater ease, significantly reducing the time and effort required to convey complex ideas. The app’s predictive capabilities were particularly beneficial, as they not only expedited the communication process but also assisted in language development by introducing a range of vocabulary and sentence structures. The choice of this app reflects the subject’s needs and preferences for tools that are uncomplicated yet powerful in their functionality and it underscores the importance of matching technological tools to the user’s capabilities and goals, ensuring that the technology serves as an aid rather than a hindrance to communication.

**Table 1** provides a summarized overview of the subject’s developmental progression with Augmentative and Alternative Communication (AAC) tools, highlighting key milestones and interventions at each age.

**Table 1:** Progression of communication milestones.

Age	Milestone/Intervention	Description
10	Introduction of Letter Board	Initial difficulties with motor coordination. Began with oversized boards featuring only eight letters to simplify communication attempts. Achieved first successful communication within a few months. Regular sessions established for dexterity and confidence enhancement.
11	Progress in Communication Skills	Significant improvement in communication. Transitioned to a full-sized letter board with all letters, allowing for a broader range of communication. Structured sessions with picture-based activities and word formation exercises continued. Family support, especially from father, crucial in reinforcing skills.
12	Transition to iPad	Transition influenced by need for more dynamic communication tool. iPad introduced with functionalities like voice output and interactive apps. An Apple keyboard was added for tactile feedback to the keys, which was crucial for the subject’s motor skills. Customized training for interface adaptation.
Post-12	Post-transition Development	Remarkable progress in academic and social communication. Active engagement in classroom activities, including quickly selecting Kahoot choices, demonstrating the practical application of AAC tools in real-world settings. Interest in science and mathematics. iPad used for complex concepts understanding and nuanced communication.

## Detailed developmental progression using AAC tools

This section explores the detailed developmental progression of our subject, focusing on his journey with Augmentative and Alternative Communication (AAC) tools. The narrative provides a comprehensive view of his growth and challenges, highlighting the pivotal role of AAC in enhancing his communication skills.

## Initial introduction of the letter board at age 10

At age 10, upon being introduced to a letter board, the subject initially struggled with motor coordination, making accurate pointing challenging. Despite these difficulties, he made his first successful attempt at communication within months, spelling out basic words and phrases such as “yes,” “no,” “more,” and “hungry.” Regular sessions aimed at improving his motor skills and confidence with the letter board included exercises for enhancing dexterity and engaging learning games.

## Notable progress in communication skills at age 11

By age 11, notable advancements were evident. The therapy sessions, now more structured, combined picture-based activities with word formation exercises to broaden his vocabulary and expressive capabilities. One notable example of his progress was forming sentences like “I want to go outside” and expressing preferences, such as “I like pasta.” His father played a critical role in this phase through daily practice, reinforcing the skills learned during therapy sessions.

## Transition to the iPad at age 12

The decision to transition to an iPad at age 12 was influenced by the subject’s growing need for a more dynamic communication tool. The iPad offered a wider range of functionalities, including voice output and interactive educational apps. The transition involved initial challenges, such as adapting to the touch interface. Customized training sessions were designed to familiarize the subject with the iPad’s interface. Gradually, he learned to navigate various apps and use the device for more complex communication. For example, he progressed from simple requests to more intricate expressions of thoughts and feelings, such as “I feel happy today because I saw my friend.”

## Post-transition development and achievements

Following the transition, the subject demonstrated significant academic and social communication strides. His active engagement in classroom activities increased, particularly in subjects like science and mathematics, facilitated by the iPad’s educational apps. Notably, his ability to express nuanced thoughts and emotions evolved remarkably such as initially communicating in brief sentences, he now articulates complex feelings and observations, such as “Why are you saying that? It is hurting this person’s feelings,” displaying a profound level of empathy and understanding.

Despite the significant strides made by the subject in utilizing AAC tools, it is important to reflect on the initial skepticism and challenges encountered. Children with apraxia, like our subject, often face perceptions of incapability in making selections or choices, which can hinder their progress. The journey to consistent pointing on the letter board, taking two years of intense work, exemplifies the necessity of perseverance, encouragement, patience and tailored support. This period of growth, however, also underscores a disheartening reality: the lack of support for AAC devices in specialized educational settings. Throughout his attendance at a specialized school, the subject encountered a lack of belief in his abilities to read or write among teachers, who showed little interest in AAC device training. This experience highlights the critical need for greater awareness and integration of AAC tools in educational environments, ensuring that children like our subject are afforded the support and belief in their potential to communicate effectively and develop autonomy.

## Discussion

The journey of the subject in this case study exemplifies the transformative potential of AAC devices in facilitating communication for individuals with limited or no verbal language. This observation is consistent with findings from Skenazy's 2022 (2) article, “How a Miracle Tool Enables Severely Autistic Kids to Communicate for the First Time,” which underscores the profound impact of AAC devices, especially letter boards, in enhancing the communicative abilities of individuals with ASD. Parallel advancements have been reported at institutions such as the Acton Academy of Eastern Long Island, where students communicate effectively using a laminated alphabetical placemat. The deployment of AAC devices, like letter boards, offers not only a communicative medium for those with fine motor challenges but also highlights their capacity to understand and engage with intricate questions and subjects. Such evidence accentuates the importance of early intervention and sustained support for individuals diagnosed with ASD and underscores the imperative to consider AAC devices as pivotal components in communication strategies.

Additionally, the findings of this case study align with recent research by Laubscher et al. (3), which underscores the profound impact of AAC devices, specifically video visual scene displays (video VSDs), for enhancing communication in individuals with ASD. Laubscher and colleagues investigated the efficacy of video VSDs as an intervention to bolster communication in children with ASD during play interactions with typically developing peers. The study observed marked improvements in communication skills among all ASD participants subjected to this intervention, even in the face of the inherent complexities associated with peer play interactions. The video VSD intervention utilized contextually relevant videos and incorporated touch-sensitive “hot spots” within the visual scene displays to streamline communication.

The clinical ramifications of this research are significant. The video VSD intervention not only facilitated communication in children with ASD but also fostered inclusivity by aiding their typically developing counterparts. This methodology treated both cohorts equitably, eliminating the necessity for one child to adopt a helper role. Moreover, the research indicated that children could extrapolate the use of video VSDs across diverse activities with limited preliminary instruction, enhancing adaptability in play and communicative methodologies. The integration of AAC devices into communication strategies, as illuminated in this case study and corroborated by Laubscher et al. (3), accentuates the transformative capacity of these tools in fostering effective communication and addressing the distinct communicative challenges encountered by individuals with ASD. Such congruence emphasizes the criticality of early intervention and sustained support for individuals with ASD. It also highlights the need for more research to test how well AAC devices, like letter boards, iPads, and video VSDs, work through detailed randomized controlled trials. By listening to the stories of those who have benefited from AAC devices, we stress the importance of providing these tools to people who might otherwise be misunderstood or overlooked.

Chapin et al. (4) also investigated the use of AAC technology, focusing on the AAC video visual scene display (VSD) method, to improve communication in preschoolers with ASD. Their findings suggest that introducing AAC interventions early can have a marked effect on communication outcomes. The AAC VSD method, utilizing tablet-based AAC apps with videos aligned to the child’s interests, led to a notable increase in communication exchanges during shared tasks. This research supports the idea that AAC tools can benefit individuals at various ages and developmental phases. Chapin et al.’s work emphasizes the advantages of early AAC interventions. Echoing our case study, it highlights the value of AAC technology in enhancing communication and social engagement for those with ASD, aiming to improve their overall well-being.

Srinivasan et al. (5) conducted a pilot study exploring the Jellow Communicator, a new AAC system tailored to teach requesting skills to young children with ASD in India. This AAC system was uniquely designed with socio-cultural considerations and a participatory approach, ensuring its relevance in varied linguistic and cultural settings. The study found that the Jellow Communicator effectively enhanced requesting skills in autistic children. Children were systematically trained to transition from low-tech AAC methods, like flashcards, to a high-tech AAC app akin to an iPad. These findings resonate with our case study, where moving from a letter board to an iPad was a pivotal advancement in the child’s communication development. Additionally, the positive feedback from caregivers in Srinivasan et al.’s study mirrors the empowerment seen in our case. The child’s ability to communicate with an iPad not only boosted his confidence but also allowed him to thrive in areas of interest, notably science and advanced mathematics.

Since its introduction in the 1990s, facilitated communication (FC) as an AAC method for those with complex communication needs has been a topic of debate. Opinions diverge on FC’s validity for individuals with significant speech, language, and communication challenges. While some advocate for FC’s effectiveness, others express reservations, pointing to potential facilitator influence on the results. Heyworth et al. (6) recently revisited the prevailing skepticism towards FC. By integrating research analysis with the experiences of autistic adults using FC, the authors suggest that past dismissals of FC might be based on outdated perspectives. They champion a renewed viewpoint that assumes communication competence in autistic individuals and considers FC as a potentially valid AAC method. As Heyworth et al. (6) note, “over 100 peer-reviewed articles support FC, with additional evidence confirming authorship, underscoring its potential validity and effectiveness for autistic individuals.”

Our case study echoes these insights, particularly as our subject’s use of a letter board as an AAC tool faced initial skepticism. Some educators and observers questioned potential external influences, speculating that the boy’s father might be guiding the communication. However, careful observations confirmed that the father merely provided physical support for the letter board. This is very shocking as typically developing children also receive support and prompting in their own development. The debate on FC underscores the need to explore various AAC methods and to assume communication competence in individuals with complex communication needs (CCN). Our case study emphasizes the value of support and belief in the abilities of individuals with ASD, mirroring the principles highlighted by Heyworth et al. It shows that the boy’s advancements in communication, including using an iPad and joining a mainstream school, were driven by his father’s steadfast belief and advocacy. This reinforces the importance of presuming competence and adopting a holistic approach to communication strategies for individuals with ASD.

Douglas et al.’s (7) study offers insights into the role of various family members in AAC interventions, encompassing parents and extended family. The research underscores the advantages of training multiple family members to aid the communication of children using AAC, advocating for a holistic approach within the family context. This view is mirrored in our case study, where the autistic child benefited from a broad support network. While his father was central, others, including a sibling, extended family, caregivers, school aides, peers, and educators, each played a distinct role in his communication journey. Together, these studies highlight the need for a collaborative and inclusive strategy in fostering communication in children with ASD, acknowledging the myriad of individuals who enhance their development and well-being.

The AAC communications with the subject also allowed him to understand what problems he had that were normal and which were related specifically to autism. The caregivers quickly realized that most of the frustrations of the subject were related to his conviction that every problem he was facing was caused by autism. Once the subject realized that he did have normal teenager issues, the irritation, tantrums and stress almost disappeared from his daily life and he was able to attend a normal school, make friends with the other students and reach a state where he declared himself “finally happy for the first time of my life”.

Literacy proficiency is pivotal for human development. Yet, individuals with limited or no speech often face underestimations of their literacy potential, leading to suboptimal outcomes (8). It’s crucial to understand that even basic reading abilities can open doors to richer communication experiences. Caron et al. (9) highlight the role of AAC technology in aiding individuals with ASD, both in communication and in developing vital literacy skills. Their study emphasizes the benefits of adapting AAC apps to include Transition to Literacy (T2L) features, which present text alongside speech when graphic symbols are selected. This approach notably boosts single-word reading skills in those with ASD. Such findings resonate with our case study, showcasing how AAC tools, like the letter board and iPad, not only foster communication but also support literacy growth.

Muharib et al. (10) conducted a study that offers insights into the efficacy of AAC technologies, specifically iPad-based speech generating devices (SGDs). A prevalent concern they addressed is the belief that introducing such AAC systems might discourage a child from verbal communication, a worry often voiced by parents. Contrary to this belief, their research found that a participant, primarily using the iPad-based SGD, eventually chose spoken language even when the SGD was accessible. This finding challenges the idea that AAC devices impede speech development. Instead, it suggests that they can both aid communication and promote spoken language in individuals with ASD. This mirrors our case study, where the participant transitioned from a letter board to an iPad-based AAC system. Notably, our participant also started using spoken words, underscoring AAC’s multifaceted benefits. This aligns with growing research that emphasizes AAC’s crucial role in enhancing communication and nurturing language development in children with ASD.

Hijab et al. (11) conducted research that sheds light on the potential of technology to bolster the social and communication skills of adults with ASD. Their study introduced the MAAN messaging app, an acronym for “Mobile Augmentative and Alternative Communication,” which is enhanced with features like text-to-speech (TTS), speech-to-text (STT), and communication symbols (CS). MAAN is designed to support individuals with communication challenges by providing a user-friendly platform that facilitates easier expression and understanding. The findings emphasize the value of such features in enhancing communication and mitigating feelings of social isolation in this demographic. This aligns with our case study, where technology, particularly the MAAN app, played a pivotal role in advancing the participant’s communication capabilities.

Another intriguing facet of this case study is the child’s acute awareness of global concerns. Using the letter board, he articulated apprehensions about environmental issues, particularly CO2 emissions and their environmental repercussions. This burgeoning environmental consciousness underscores his deep regard for global challenges and the planet’s health. These observations highlight that AAC tools do more than facilitate academic and personal communication; they enable individuals to engage with and address societal matters. The ability to express empathy and champion causes further illustrates the expansive influence of AAC devices on autistic people.

The body of evidence supporting the efficacy of AAC devices is substantial, highlighting their transformative potential for individuals with communication challenges. Despite this, the journey of our subject highlights a stark contrast between evidence-based support for AAC and its acceptance and implementation in specialized educational settings. This discrepancy underscores the critical need for increased awareness, training, and systemic support to bridge the gap between AAC’s proven benefits and its mainstream adoption.

Our case study further emphasizes the necessity of a patient, individualized approach to AAC implementation and the importance of presuming competence in every individual, regardless of his or her communication challenges. The narrative of our subject’s journey with AAC devices not only illustrates the significant advancements made possible through tailored interventions and dedicated support but also highlights the hurdles faced due to prevailing misconceptions and institutional barriers. The challenges encountered in securing acknowledgment and support for AAC use in specialized schools reflect a broader issue within educational and therapeutic environments. This disconnect underscores an urgent call for action to integrate AAC more fully into mainstream educational strategies. Increased exposure to AAC success stories and further research can play a pivotal role in shifting perceptions, advocating for a more inclusive approach that recognizes the capabilities and potential of all individuals.

In conclusion, our investigation contributes to the growing body of literature advocating for the widespread implementation of AAC devices, strengthening the case for increased empirical and practical evidence to support AAC utilization. The emphasis on patience, individualized intervention, and the core assumption of innate competence in each person establishes critical directives for educators, therapists, and policy developers. This approach is imperative for the formulation of more inclusive communication and educational policies that ensure comprehensive support and recognition of all individuals’ communicative abilities. By adopting these principles, we can advance towards a future in which AAC devices are not merely acknowledged for their capabilities but are systematically incorporated into communication and educational frameworks, guaranteeing that every individual’s communicative needs are met.

## Transition from special needs education to mainstream schooling and subsequent academic success

### Background and initial assessment

The beginning of the subject’s educational path was marked by challenging assessments. A psychologist at the special needs school, relying heavily on traditional observation methods, concluded that the subject exhibited characteristics of severe Intellectual Disability (ID) within the autism spectrum disorder framework. This evaluation, largely focused on the subject’s nonverbal cognitive and adaptive functioning, suggested significant delays. The assessment, however, was conducted in the absence of crucial elements that had proven to support the subject’s ability to communicate and learn effectively—specifically, without the presence of his father and devoid of the use of an iPad, a medium through which the subject could express himself and interact with his educational content more effectively. This initial assessment painted a grim picture of the subject’s potential, categorizing him within a framework that emphasized limitations over capabilities. The conclusions drawn were stark, framing the subject’s cognitive and adaptive skills within a narrative of severe limitations. This perspective was shaped without considering the supportive interventions and technologies that could unlock the subject’s abilities. The focus was on the here and now of the subject’s capabilities, assessed under conditions that did not reflect his everyday environment or the support network that facilitated his learning and communication. The school’s approach, while standard for assessing intellectual and adaptive functioning failed to account for the dynamic nature of autism spectrum disorders and the profound impact that tailored support and adaptive communication methods could have on a child’s development. This oversight set the stage for a journey filled with low expectations of the subject’s potential, overlooking the difference that a supportive environment could make in unlocking his true capabilities.

### Neuropsychological findings - recognition of capabilities and educational implications

In contrast to the school’s evaluation, a home-based assessment showed a very different picture. A neuropsychologist, utilizing an iPad and conducting the evaluation in the reassuring presence of the subject’s father, recognized the child’s latent abilities. This supportive setting allowed for a clear demonstration of the subject’s cognitive strengths and academic potential, revealing that when provided with appropriate tools and support, the subject could excel far beyond the limited expectations previously set for him. The findings of the neuropsychological evaluation at home unveiled the subject’s verbal comprehension, strong working memory, and reasoning capabilities. These cognitive strengths, which had been overshadowed in the less accommodating special needs school setting due to communication challenges and heightened anxiety, came to light in an environment where the subject felt secure and supported. The psychologist’s report noted the subject’s adeptness with complex orthography, remarkable psychometric test performance, and a keen interest in sophisticated fields such as astrophysics, revealing a high level of intellectual curiosity and potential for academic success. The neuropsychological assessment highlighted the need for educational strategies tailored to the subject’s unique cognitive profile and emotional needs, which included behavioral therapy to address anxiety and tantrums, physical therapy to improve motor skills, and speech therapy to support communication. Furthermore, the evaluation suggested that the subject’s educational plan be adapted to include one-on-one tutoring and a curriculum that aligns with his interests and strengths. This evaluation was instrumental in the subject’s educational transformation as it underlined the importance of a holistic approach to education that considered the full spectrum of a child’s complexities.

### Progress in mainstream schooling

These insights lead to a shift in the educational approach for the subject, ultimately resulting in his transfer to a mainstream school that provided an enriched learning environment which marked a turning point for the subject. The new school environment, tailored to his unique needs, offered a nurturing setting for his innate abilities to flourish and expand and as a result, the subject achieved impressive academic success, with his report cards from the mainstream school reflecting excellence across various subjects which was a stark contrast to the misconceptions about his cognitive abilities that had once defined his educational assessments.

### Analysis of report cards

The educational trajectory of the subject shows a significant change in academic performance when transitioning from a special needs to a mainstream school setting. In the special needs school setting, the subject’s assessment results (**Table 2**) were focused on competency development and the level of support required to achieve these competencies which included communicative abilities, application of information, interactions with others, methodical behavior, and safety protocols stated that the subject required “Frequent Support” in most areas, with “Communicates” and “Acts Methodically” necessitating only “Occasional Support”. Also, per the curriculum-specific skills within the special needs framework (**Table 3**), the levels of support required by the subject were significant, with “Constant Support” needed in oral communication and writing, and “Frequent Support” in reading, number sense, and climate understanding. On the other hand, the subject’s performance in the mainstream school depicted a very different academic profile (**Table 4**) where the subject’s grades were exemplary, with scores ranging from the high 80s to high 90s across two terms and were consistently above the group average, indicating not just competence but academic excellence and a capacity to exceed peer-level performance. The comparison between the two educational settings suggests that the mainstream school environment, with its enriched curriculum and individualized support allowed the subject to excel beyond the expectations previously set by the special needs assessment. The subject’s remarkable grades and recognition on the Honor Roll dispel any doubts about his abilities as the subject has maintained a high level of academic performance with a general average score of 91 in both Term 1 and Term 2.

**Table 2:** Special needs school - general competencies - assessment results.

Competency	Support Level	Competency Details
Communicates	Occasional Support	This competency is centered on the student’s ability to share emotions, comprehend messages, and engage with others.
Uses information	Frequent Support	Involves the application of diverse resources within the educational setting to steer one’s conduct.
Interacts with others	Frequent Support	Encourages constructive interactions, awareness of others’ needs, adherence to guidelines, and accountability for personal actions.
Acts Methodically	Occasional Support	Students strive to follow varied procedures throughout the day to reach defined goals, enhancing independence through systematic actions.
Acts in a safe manner	Frequent Support	Incorporates the adoption of secure behaviors and adherence to safety protocols.

Reporting term: term 1 starting: 2020-08-30 ending: 2021-01-22.

**Table 3:** Special needs school – curriculum specific skills - assessment results (12).

Subject Area	Skill Focus	Support Level	Learning Objective
English Language Arts	Oral Communication	Constant Support	Expresses a message that can be understood.
English Language Arts	Reading	Frequent Support	Knowledge of pictogram and word meanings: Knows the pictograms used in the classroom.
English Language Arts	Writing	Constant Support	Trace shapes, letters, and numbers.
Mathematics	Number Sense and Use	Frequent Support	Counts objects from 1 to 9.
Mathematics	Number Sense and Use	Constant Support	Identifies numbers from 0 to 9.
Science	Climate	Frequent Support	Recognizes different weather conditions.
Science	Climate	Frequent Support	Associates weather conditions with appropriate items of clothing.

Reporting term: term 1 starting: 2020-08-30 ending: 2021-01-22.

**Table 4:** Regular mainstream school – curriculum specific skills - assessment results (13).

Subject	Competency	Subject Mark (Term 1)	Subject Mark (Term 2)	Group Average (Term 1)	Group Average (Term 2)
English Language Arts	Uses language to communicate and learn, reads and listens to texts, produces spoken, written and media texts	90	91	85	86
French, Second Language	Communicates in French, understands oral and written texts in French, produces oral and written texts in French	96	97	91	89
Mathematics	Solves a situational problem, uses mathematical reasoning	87	89	87	91
Science and Technology	Practical, Theory	92	86	76	74
Geography		98	98	76	71
History and Citizenship Education		90	89	80	73
Music		95	92	82	83
Ethics and Religious Culture		91	88	74	75
Study Methods		95	98	79	76

Results term 1 - starting 2023-08-23 ending 2023-11-03 - term 2 - starting 2023-11-06 ending 2024-02-02.

## Recommendations

The insights derived from the subject’s educational journey highlight the need for a shift in the assessment and education of individuals with ASD. It is imperative that educators and practitioners re-evaluate the efficacy of traditional assessment methods which may not capture the true potential of individuals with ASD, often failing to accommodate the unique ways in which these individuals may express their capabilities. An alternative assessment strategy that employs AAC tools, alongside a supportive presence, could serve as a more accurate indicator of their abilities. Educational plans must also be adjusted to cater to the individualized cognitive and emotional needs of those with ASD and they should not be static but rather dynamic addressing the specific interests and strengths of each student. This personalized approach is pivotal in fostering an environment where students with ASD can thrive academically and socially. In fact, the integration of AAC tools into educational settings should be considered best practice, not an exception as they are invaluable for facilitating communication and cognitive development, and their use should be widespread in both special needs and mainstream classrooms. For those students transitioning from special needs to mainstream education, it is essential to provide a structured and supportive transition process in order to mitigate the challenges posed by new academic and social settings, ensuring students can navigate these environments successfully.

Furthermore, the transformation witnessed in the subject’s academic performance highlights an urgent call for further research as presently there is a significant gap in understanding the long-term academic trajectories of individuals with ASD who utilize AAC tools. Rigorous research, including randomized controlled trials, is necessary to evaluate the effectiveness of these tools across various ASD contexts systematically. The subject’s transition from a special needs school to securing an impressive 91% average and attaining Honor Roll status after one year in a mainstream school illustrates the significant influence that customized assistance and AAC tools possess in the educational advancement of students with ASD. This transformation in academic results also shows the positive impact of treating the individual as a student with potential rather than a disabled limited child. This academic achievement not only reflects the subject’s individual dedication and skill but also acts as an argument for the reevaluation of educational methodologies for such students. As we move forward, in delivering interventions for autistic children, it is important that professionals begin with a presumption of competence. Presuming competence sets a tone of respect and expectation that can significantly influence the child’s performance and engagement. Autistic children especially those with apraxia, often face undue skepticism, which may hinder their ability to demonstrate their true abilities. Therefore, we advocate for a belief in their potential for communication and learning.

Equally important is the context in which evaluations and assessments are conducted. It is critical to create “win” conditions for the child—a supportive environment where the child feels understood and believed in. This study observed stark differences in the subject’s performance when assessed under stressful conditions at school versus a supportive environment at home. In the latter setting, where the child was at ease and in the presence of familiar and supportive individuals, in this case the subject’s father, the assessments conducted by a neuropsychologist over multiple sessions with the subject’s preferred AAC device (iPad) revealed a more accurate representation of his abilities. This underscores the need for assessments to be conducted in environments that are conducive to the child’s best performance, free from stressors that may inhibit their ability to engage fully. Furthermore, patience and adaptability in the application of AAC tools are key. The subject’s journey from using an oversized letter board with limited letters to a full letter board, and eventually to an iPad with an external keyboard, exemplifies a gradual approach to AAC introduction as the progression was not immediate but spanned four years, highlighting the need for sustained commitment and patience from both professionals and caregivers. The eventual use of AAC tools by the subject in interactive classroom activities, such as Kahoot, illustrates the practical application and integration of these tools into daily life and education.

Given the transformative potential of AAC devices demonstrated in this study, healthcare professionals should actively incorporate tools such as letter boards and iPads into their communicative strategies with autistic children as such devices have shown efficacy in improving the articulation of thoughts and engaging in academic and social interactions. Moreover, this case study reinforces the imperative for further research, as outlined in our abstract. Conducting randomized controlled trials is crucial to evaluate the efficacy of AAC devices across a broader autistic population as rigorous scientific validation will help dispel doubts and substantiate the broader application of these communication aids.

## Conclusion

This case study underlines the transformative power of AAC devices, especially letter boards, in fostering effective communication for autistic people with limited or no verbal language. These tools offer a beacon of hope for families and individuals grappling with communication barriers, underscoring the significance of early intervention and sustained support. By integrating AAC devices into therapeutic strategies, professionals can unveil the latent potential in autistic individuals, enabling them to engage more fully with society. The testimonials of those who have thrived with AAC devices accentuate the urgency of availing these tools to those who might otherwise be misinterpreted or overlooked. The adoption of AAC technology, as illustrated in this study, combined with emerging innovations like the MAAN app, displays the potential of contemporary assistive technologies, which promise to elevate individuals with ASD by improving their communicative abilities and fostering societal integration and acceptance. In essence, this study advocates for a holistic and inclusive approach to AAC solutions for the ASD community and patience while motor skills need improvement. By acknowledging communication complexities and maintaining an assumption of competence, we pave the way for a more inclusive approach in supporting the autism community’s communicative needs.

## Data availability statement

The original contributions presented in the study are included in the article/supplementary material. Further inquiries can be directed to the corresponding author.

## Ethics statement

Written informed consent was secured from the appropriate parties directly involved in the study for its publication, ensuring compliance with ethical standards and respect for participant autonomy. Written informed consent was obtained from the participant/patient(s) for the publication of this case report.

## Author contributions

DM: Writing – original draft, Writing – review & editing.
